# Local and Systemic RAGE Axis Changes in Pulmonary Hypertension: CTEPH and iPAH

**DOI:** 10.1371/journal.pone.0106440

**Published:** 2014-09-04

**Authors:** Bernhard Moser, Anna Megerle, Christine Bekos, Stefan Janik, Tamás Szerafin, Peter Birner, Ana-Iris Schiefer, Michael Mildner, Irene Lang, Nika Skoro-Sajer, Roela Sadushi-Kolici, Shahrokh Taghavi, Walter Klepetko, Hendrik Jan Ankersmit

**Affiliations:** 1 Department of Thoracic Surgery, Division of Surgery, Medical University Vienna, Vienna, Austria; 2 Christian Doppler Laboratory for the Diagnosis and Regeneration of Cardiac and Thoracic Diseases, Medical University Vienna, Vienna, Austria; 3 Department of Cardiac Surgery, University of Debrecen, Debrecen, Hungary; 4 Department of Pathology, Medical University Vienna, Vienna, Austria; 5 Department of Dermatology, Medical University Vienna, Vienna, Austria; 6 Department of Internal Medicine II, Division of Cardiology, Medical University Vienna, Vienna, Austria; Vanderbilt University Medical Center, United States of America

## Abstract

**Objective:**

The molecular determinants of chronic thromboembolic pulmonary hypertension (CTEPH) and idiopathic pulmonary arterial hypertension (iPAH) remain poorly understood. The receptor for advanced glycation endproducts (RAGE) and its ligands: HMGB1 and S100A9 are involved in inflammatory disorders. We sought to investigate the role of the RAGE axis in patients with CTEPH undergoing pulmonary endarterectomy (PEA), iPAH undergoing lung transplantation (LuTX). The high pulmonary vascular resistance in CTEPH/iPAH results in pressure overload of the right ventricle. We compared sRAGE measurements to that of patients with aortic valve stenosis (AVS) – pressure overload of the left ventricle.

**Methods:**

We enrolled patients with CTEPH(26), iPAH(15), AVS(15) and volunteers(33). Immunohistochemistry with antibodies to RAGE and HMGB1 was performed on PEA specimens and lung tissues. We employed enzyme-linked immunosorbent assays to determine the concentrations of sRAGE, esRAGE, HMGB1 and S100A9 in serum of volunteers and patients with CTEPH, iPAH, AVS before and after PEA, LuTX and aortic valve replacement (AVR).

**Results:**

In endarterectomised tissues from patients with CTEPH RAGE and HMGB1 were identified in myofibroblasts (α-SMA^+^vimentin^+^CD34^−^), recanalizing vessel-like structures of distal myofibrotic tissues and endothelium of neointima. RAGE was differentially expressed in prototypical Heath Edwards lesions in iPAH. We found significantly increased serum concentrations of sRAGE, esRAGE and HMGB1 in CTEPH. In iPAH, sRAGE and esRAGE were significantly higher than in controls. Serum concentrations of sRAGE were significantly elevated in iPAH(p<0.001) and CTEPH(p = 0.001) compared to AVS. Serum sRAGE was significantly higher in iPAH compared to CTEPH(p = 0.042) and significantly reduced in AVS compared to controls(p = 0.001). There were no significant differences in sRAGE serum concentrations before and after surgical therapy for CTEPH, iPAH or AVS.

**Conclusions:**

Our data suggest a role for the RAGE pathway in the pathophysiology of CTEPH and iPAH. PEA improves the local control of disease but may not influence the systemic inflammatory mechanisms in CTEPH patients through the RAGE pathway.

## Introduction

Pulmonary hypertension (PH) is currently defined as a hemodynamic and pathophysiological condition with a mean pulmonary artery pressure (PAP*mean*) of ≥25 mmHg at rest. The European Society of Cardiology (ESC) and European Respiratory Society (ERS) have classified these conditions into six groups. Pulmonary arterial hypertension (PAH, group 1) can be the result of a wide array of underlying diseases. The entity idiopathic pulmonary arterial hypertension (iPAH, group 1.1) is used if no underlying causative disease can be diagnosed. The increase in pulmonary vascular resistance (PVR) is related to different mechanisms, including vasoconstriction, proliferative and obstructive remodeling of the pulmonary vessel wall, inflammation and thrombosis. The pathology of idiopathic pulmonary arterial hypertension affects the small distal pulmonary arteries (PAs) with a diameter less than 500 µm. Typical findings are hypertrophy of the media, intimal proliferative and fibrotic changes, thickening of the adventitia with perivascular inflammatory infiltrates, complex and thrombotic lesions [1]. A widely used pathological grading system for pulmonary arterial changes in hypertensive pulmonary vascular disease was published by Heath and Edwards in 1958 [2], [3].

In stark contrast to iPAH, the characteristic pathology of chronic thromboembolic pulmonary hypertension (CTEPH, group 4) is remodeled central and proximal PAs. Organized thrombotic formations build the luminal lining of the PA vessel wall replacing physiological intima [1]. Endarterectomized tissues from patients with CTEPH show vessel-like structures in material obtained from distal areas, whereas proximal material is characterized by lower cell density and sometimes the accumulation of fresh thrombotic material [4].

As described above, iPAH and CTEPH are progressive diseases of the distal (iPAH) and proximal (CTEPH) pulmonary vessels leading to increased PVR and PAP. In the further course of these diseases right ventricular dysfunction and ultimately right ventricular failure is the leading cause of death. The prognosis of patients is associated to right ventricular performance measures, such as cardiac index and right atrial pressure. Right heart failure is caused by pressure overload of the right ventricle. The increase in wall stress leads to increased wall thickness by muscular hypertrophy (increase in cell size by addition of sarcomeres). However, the chronic exposure to high RV pressures results in RV dilation with a decrease in contractile forces. Pathological inflammatory responses, oxidative stress and humoral responses may further promote right heart failure [5]. The consequences of RV failure are intractable ascites, renal impairment, malnutrition and immobility [5], [6]. Pharmacological therapy effective in PAH did not prove to benefit patients with CTEPH [7]. Surgical therapy for selected patients with CTEPH is pulmonary endarterectomy (PEA) [8]. Patients with CTEPH who are not amenable to PEA and patients with iPAH are possible candidates for lung transplantation (LuTX).

The overexpression of cytokine cascades may contribute to the progression of heart failure - “cytokine hypothesis” for heart failure [9]. Most of our knowledge on neurohormonal and cytokine signaling, oxidative stress, inflammation, ischemia, and cell death which may contribute to RV dilatation and failure is inferred from research on left sided heart failure [10]. In patients with chronic left-sided heart failure increased serum concentrations of proinflammatory cytokines, such as tumor necrosis factor-alpha (TNF-alpha), interleukin(IL)-1, and IL-6 correlate with clinical and hemodynamic parameters of disease severity [11], [12]. A S100 protein family member, S100A8/A9, has recently been shown to activate cardiac fibroblasts to initiate angiotensin II-induced hypertension cardiac injury [13]. Recently, increased left ventricular hypertrophy (LVH) and diastolic dysfunction was demonstrated in chronic uremic mice with transgenic expression of the human S100/Calgranulin gene cluster containing the genes and regulatory elements for S100A8, S100A9, and S100A12. S100/calgranulin-mediated inflammation induced fibroblast growth factor 23 (FGF23) in cardiac fibroblasts which in a paracrine manner may mediate LVH and diastolic dysfunction [14]. The role of biomarkers in pulmonary hypertension has been reviewed recently [15].

The Receptor for Advanced Glycation Endproducts (RAGE), a transmembrane receptor, is a member of the immunoglobulin superfamily of receptors that interacts with different ligands. Since its first characterization in 1992 where a high basal expression in the lung was shown [16] a vast literature around this receptor, its lung and vascular biology and pathology has evolved [17], [18], [19], [20]. First, advanced glycation endproducts (AGEs) were identified as ligands [16]. Next, RAGE was identified as a cell surface receptor for S100/Calgranulins amplifying chronic cellular activation and tissue injury [21]. Further ligands were detected later: high-mobility group box 1 (HMGB1) - also known as amphoterin [22], Mac-1 and others. The current view that RAGE - RAGE-ligand interaction augments pro-inflammatory pathways is supported by the detection of RAGE and RAGE ligands in tissues of various disease processes, such as arteriosclerosis [18], diabetes [23], glomerulosclerosis [24], periodontal disease [25], arthritis [26], transplantation [27] and other chronic inflammatory disorders.

The extracellular soluble form of the receptor (sRAGE) can be detected in serum of patients [28]. Proteolytic shedding of RAGE by metalloproteinases has been described [29], [30]. It functions to bind ligands and thereby blocks interaction with and activation of cell surface RAGE. An increased concentration of RAGE-ligands leads to the formation of circulating sRAGE-ligand complexes. An increasing occupation of sRAGE leads to lower concentration of free sRAGE in serum and therefore directs to increased surface RAGE-ligand interaction and possibly to a boost in inflammation. Nevertheless, it is currently unknown if high plasma/serum concentrations of sRAGE can be interpreted as protection against chronic inflammation or correlated with high levels of ongoing inflammation [29]. Currently available tools to measure sRAGE don’t separate between sRAGE-ligand complexes and free sRAGE. There is an alternative splice variant of the RAGE gene, called endogenous secretory RAGE (esRAGE) that is actively secreted. [31], [32]. In conclusion, neither the function nor the source of sRAGE in human physiology is known. Expression profiling for esRAGE in multiple human organs and RAGE in human thymus has recently been performed [33], [34]. Plasma/serum concentrations of sRAGE in diabetes mellitus type 2 and coronary artery disease have been studied with conflicting findings [35], [36].

The RAGE ligand HMGB1 is a non-histone chromosomal protein which functions as a DNA chaperone. The molecule is composed of two homologous DNA binding domains and an acidic tail. Different binding domains for its receptors: RAGE, Toll-like receptor 4 (TLR4) and a p53 transactivation domain have been identified. Once HMGB1 is released from the cell it acts as a signaling molecule, namely a damage-associated molecular pattern molecule (DAMP) [37], [38].

There is a myriad of studies (mostly animal models) implicating a role for RAGE and its ligands in the pathogenesis of vascular disease. Most of these studies have investigated the systemic vessels. Studies in mice with diabetic atherosclerosis showed that treatment with murine sRAGE suppressed the development of accelerated diabetic atherosclerosis in a dose-dependent manner [39], [40].

The hypothesis that RAGE could be a key player in pulmonary hypertension was inferred from evidenced based reviews of the scientific literature [41]. The RAGE – RAGE-ligand axis might drive the inflammatory changes in the walls of pulmonary vessels (macro- and microangiopathy) in a similar way as has been shown for the systemic vasculature. A role for RAGE in human pulmonary artery smooth muscle cells (hPASMCs) of patients with idiopathic pulmonary arterial hypertension (iPAH) and in *in vivo* animal models of monocrotaline- and Sugen-induced PAH was recently described [42]. Further, HMGB1 was shown to contribute to PH via a Toll-like receptor 4 (TLR4)-dependent mechanism in a murine model of chronic hypoxia (CH)-induced PH [43].

In a mouse model of hypobaric hypoxia (10% O_2_)-induced PH treatment with sRAGE was protective against increases in RV pressure but did not affect distal pulmonary vascular remodeling. In vitro the administration of sRAGE modulated vasoreactivity of intralobar pulmonary arteries from hypobaric hypoxic mice and further enhanced hypoxia-induced proliferation of Chinese hamster lung fibroblasts [44].

We sought to investigate a possible role of RAGE and HMGB1 in diseased main to segmental pulmonary arteries of patients with CTEPH undergoing PEA and small PAs (<500 µm) in iPAH patients undergoing lung transplantation. Moreover, we hypothesized that systemic inflammatory changes pertaining to RAGE and RAGE ligands (HMGB1, S100A9) can be measured in patients with CTEPH and iPAH. We compared systemic measurements of CTEPH and iPAH patients to those of patients with aortic valve stenosis (AVS). We aimed to filter out changes specific to PH, a disease characterized by pressure overload of the right ventricle in comparison to a disease that inflicts pressure overload on the left ventricle, such as AVS. Lastly, we sought to investigate the effects of surgical therapy, such as PEA, LuTX and AVR on systemic inflammation.

## Materials and Methods

### Ethics Statement

Ethical approval was obtained from the Medical University Vienna review board on human research. Written informed consent was obtained from all patients and volunteers participating in this study.

### Definitions

PH is defined as an increase in mean pulmonary arterial pressure (PAP) ≥25 mmHg at rest as assessed by right heart catheterization [1]. CTEPH is defined by the following observations after ≥3 months of effective anticoagulation: (1) mean PAP≥25 mmHg with a pulmonary capillary wedge pressure (PCWP) ≤15 mmHg; and (2) at least one (segmental) perfusion defect detected by lung scanning, multi-detector computed tomography angiography or pulmonary angiography [45].

iPAH is a clinical condition characterized by the presence of precapillary PH in the absence of other causes of precapillary PH [1].

### Subjects

We prospectively enrolled 26 patients with CTEPH undergoing PEA, 15 patients with iPAH undergoing lung transplantation, 15 patients with severe aortic stenosis undergoing aortic valve replacement and 33 healthy control subjects between 2010 and 2014. PEA and LuTX surgery were carried out at the department of thoracic surgery, Medical University Vienna, aortic valve replacement (AVR) surgery at the department of cardiac surgery, University Debrecen. The diagnosis of CTEPH and indication for PEA surgery was established by teams of specialists in the diagnosis and treatment of patients with pulmonary hypertension, CTEPH and lung transplantation in every case. All patients with CTEPH were classified to have type 2 disease, intimal thickening and fibrosis proximal to the segmental arteries, according to the intraoperative classification system [8]. The diagnosis of AVS and indication for AVR surgery was established by teams of specialists in cardiology and cardiac surgery in every case. Patient characteristics are given in Tables 1–3.

**Table 1:** The basic characteristics of patients with CTEPH and controls (healthy volunteers) are listed.

	CTEPH (n = 26)	Controls (n = 33)	*p* value
**Age** *in years*	51.9 (56.2) ±14.7 (2.9), [31–75]	54.2 (54.0) ±15.2 (2.6), [30–83]	0.828
**F:M ratio** *n (%)*	9∶17 (34.6∶65.4)	12∶21 (36.4∶63.6)	0.985
**PAP** *_mean_ [mmHg]*	52.9 (52.0) ±14.6 (2.9), [32–90]		
**PVR** *[dynes·s^−1^·cm^−5^]*	787.2 (757.0) ±386.6 (75.8), [281–1646]		
**CI** *[l/min/m^2^]*	4.4 (4.5) ±0.99 (0.19), [2.2–5.4]		
**sRAGE** *[pg/ml]*	467.2 (331.8) ±370.4 (72.6), [105.0–1461.6]	198.6 (142.8) ±162.9 (28.3), [6.4–807.6]	0.001
**esRAGE** *[pg/ml]*	703.7 (610.8) ±309.3 (63.1), [170.0–1370.0]	414.5 (378.8) ±177.1 (31.8), [180.0–790.0]	<0.001
**S100A9** *[µg/ml]*	2.1 (0.7) ±3.9 (0.8), [0.3–18.2]	0.7 (0.6) ±0.5 (0.09), [0.2–2.1]	0.064
**HMGB1** *[pg/ml]*	1141.1 (865.8) ±865.6 (173.1), [226.5–3584.8]	464.3 (411.3) ±371.1 (66.6), [0–1818.4]	0.001

Reported is mean (median) ± standard deviation (standard error mean), [range].

*CTEPH* chronic thromboembolic pulmonary hypertension, *n* number of patients, *F:M* ratio female to male ratio, *PAP_mean_* mean pulmonary artery pressure, *PVR* pulmonary vascular resistance; *CI* cardiac index, *sRAGE* soluble receptor for advanced glycation endproducts, *esRAGE* endogenous secretory receptor for advanced glycation endproducts, *S100A9* member of S100 family of Ca^+^ binding proteins, *HMGB1* high mobility group box1.

**Table 2:** The basic characteristics of patients with iPAH and controls (healthy volunteers) are listed.

	iPAH (n = 8)	Controls (n = 11)	*p* value
**Age** *in years*	36.6 (37.4) ±9.9 (3.5), [21]–[50]	36. 4(33.0) ±12.3 (3.7), [22]–[62]	0.963
**F:M ratio** *n (%)*	8∶0 (100∶0)	10∶1 (90.9∶9.1)	0.381
**PAP** *_mean_ [mmHg]*	54.5 (57.5) ±20.0 (10.0), [28–75]		
**PVR** *[dynes·s^−1^·cm^−5^]*	1425.0 (1505.0) ±582.1 (291.0), [706–1984]		
**CI** *[l/min/m^2^]*	3.7 (3.8) ±0.4 (0.1)		
**sRAGE** *[pg/ml]*	743.7 (401.9) ±672.9 (254.3), [123.9–1861.7]	195.5 (130.8) ±130.9 (39.5), [51.0–441.1]	0.017
**esRAGE** *[pg/ml]*	1391.1 (972.9) ±1073.2 (379.4), [280.0–3110.0]	423.2 (399.4) ±197.1 (59.4), [210.0–750.0]	0.009
**S100A9** *[µg/ml]*	1.4 (0.8) ±1.7 (0.6), [0.5–5.6]	0.9 (0.7) ±0.5 (0.1), [0.4–2.1]	0.374
**HMGB1** *[pg/ml]*	1419.4 (845.2) ±1614 (610.1), [381.5–5020.9]	415.1 (410.6) ±207.0 (65.5), [80.6–833.3]	0.067

Reported is mean (median) ± standard deviation (standard error mean), [range].

*iPAH* idiopathic pulmonary arterial hypertension, *n* number of patients, *F:M* ratio female to male ratio, *PAP_mean_* mean pulmonary artery pressure, *PVR* pulmonary vascular resistance; *CI* cardiac index, *sRAGE* soluble receptor for advanced glycation endproducts, *esRAGE* endogenous secretory receptor for advanced glycation endproducts, *S100A9* member of S100 family of Ca^+^ binding proteins, *HMGB1* high mobility group box1.

**Table 3:** Clinical characteristics of patients with CTEPH before and after PEA, iPAH before and LuTX, AVS before and after AVR.

CTEPH (n = 20)	Before PEA	After PEA	p value
**sRAGE** *[pg/ml]*	743.29 (582.90) ±117.62 (26.98), [125.33–2085.55]	688.70 (609.14) ±396.60 (88.68), [141.84–1712.88]	0.724
**Age** *in years*	60.24 (63.99) ±12.99 (2.91), [31–78]		
**F:M ratio** *n (%)*	5∶15		
**PAP_mean_** *[mmHg]*	53.53 (53.00) ±15.26 (3.50), [28.00–90.00]	27.42 (27.00) ±6.05 (1.39), [18.00–39.00]	<0.001
**PVR** *[dynes·s^−1^·cm^−5^]*	701.27 (690.00) ±277.95 (67.41), [329.00–1257.00]	265.37 (248.00) ±117.62 (26.98), [88.00–539.00]	<0.001
**CI** *[l/min/m^2^]*	1.92 (2.12) ±1.13 (0.25), [0.00–4.20]	2.08 (2.20) ±1.42 (0.33), [0.00–3.86]	0.542
**iPAH** (n = 7)	**Before LuTX**	**After LuTX**	
**sRAGE** *[pg/ml]*	1216.05 (1315.27) ±564.95 (213.53), [326.47–1835.31]	772.83 (519.91) ±694.85 (262.63), [125.95–1880.66]	0.168
**Age** *in years*	33.43 (34.00) ±6.23 (2.35), [21]–[40]		
**F:M ratio** *n (%)*	5∶2		
**PAP** *_mean_ [mmHg]*	82.57 (77.00) ±17.63 (6.66), [54.00–103.00]	22.57 (22.00) ±3.41 (1.29), [19.00–29.00]	<0.001
**PVR** *[dynes·s^−1^·cm^−5^]*	1733.60 (1800.00) ±200.71 (89.76), [1452.00–1984.00]		
**CI** *[l/min/m^2^]*	1.99 (2.00) ±0.27 (0.11), [1.70–2.30]	4.27 (4.50) ±0.59 (0.34), [3.60–4.70]	0.074
**AVS** (n = 15)	**Before AVR**	**After AVR**	
**sRAGE** *[pg/ml]*	260.26 (216.04) ±171.40 (44.26), [104.01–808.26]	274.45 (221.26) ±199.24 (51.44), [83.91–874.58]	0.804
**Age** *in years*	65.23 (64.50) ±10.31 (2.66), [44–86]		
**F:M ratio** *n (%)*	6∶9		
**PAP_mean_** *[mmHg]*	18.87 (19.00) ±4.94 (1.28), [9.00–28.00)		
**Mean grad**	47.47 (43.00) ±16.22 (4.19), [29.00–84.00]		
**Vmax** *m/s*	4.47 (4.30) ±0.73 (0.19), [3.80–6.20]		
**AVA**	0.76 (0.80) ±0.21 (0.06), [0.30–1.00]		
**Controls** (n = 28)			
**sRAGE** *[pg/ml]*	567.80 (446.78) ±329.09 (62.19) [122.48–1355.13]		
**Age** *in years*	58.50 (62.00) ±20.72 (3.92) [30–91]		
**F:M ratio** *n (%)*	12∶16		

Reported is mean (median) ± standard deviation (standard error mean), [range].

*CTEPH* chronic thromboembolic pulmonary hypertensioņ *PEA* pulmonary endarterectomy, *iPAH* idiopathic pulmonary arterial hypertension, *LuTX* double lung transplantation, *AVS* aortic valve stenosis; *AVR* aortic valve replacement, *Mean grad* mean transvalvular pressure gradient, *Vmax* maximum aortic stenosis jet velocity, *AVA* aortic valve area, *F:M* ratio female to male ratio, *PAP_mean_* mean pulmonary artery pressure, *PVR* pulmonary vascular resistance; *CI* cardiac index, *sRAGE* soluble receptor for advanced glycation endproducts, *n* number of patients.

None of the control subjects studied had any evidence or suspicion of any form of pulmonary hypertension, autoimmune disease, malignancies or infectious conditions at the time of entry into this study. None of the volunteers received anticoagulants, prostaglandins, immunosuppressant therapy or any other type of prescribed medication.

### Human tissue and serum sample collection

Fresh tissues (PEA specimens, pulmonary arteries and lung tissues) were harvested at the time of PEA (from patients with CTEPH), lung transplantation (from patients with iPAH, CF and COPD) and video-assisted thoracoscopic surgery for recurrent primary spontaneous pneumothorax (otherwise healthy individuals). Histological diagnoses and classification of iPAH in this study was routinely performed at the clinical institute of pathology at the Medical University Vienna.

Serum samples were centrifuged within 60 minutes of collection and stored at −80°C until analysis. In CTEPH the first results from serum analysis were obtained in 26 patients compared to 33 controls (table 1). For the next step we collected serum samples 10 days after (12 patients) and 1 year after PEA (9 different patients; summarized in table 3). Similarly, in iPAH the first result was obtained in serum of 8 patients compared to controls (table 2). In the next step we collected serum samples of 7 different patients with iPAH before and 3 weeks after lung transplantation (table 3). A total of 15 patients with iPAH were included in the study. Age- and sex-matched controls used for subset analysis are part of the whole control pool. Serum samples were collected before and 10 days after AVR (table 3). All postoperatively collected serum samples stem from patients with an uneventful postoperative course.

### Immunohistochemistry

Formaldehyde-fixed and paraffin-embedded human PEA specimens, lung tissues and PAs were prepared according to routine protocols of the clinical institute of pathology. Briefly, sections 2 µm in thickness, were baked for 1 hour at 55°C, deparaffinized in three xylenes and rehydrated in ethanol as follows: 2×100%, 1×95%, 1×90%, and 1×70%, followed by PBS. Antigen retrieval was performed by boiling slides at 600 watt (3×5 min) in a microwave oven using citrate buffer at pH 6.0 (Target Retrieval Solution, Dako, USA). Endogenous peroxidase activity was blocked by applying hydrogen peroxide 0.3%. Sections were incubated with 2% bovine serum albumin or blocking serum of the same species as the biotinylated secondary antibody to deplete unspecific protein-protein interactions. Sections were stained using affinity-purified polyclonal goat anti-human RAGE IgG (R&D Systems, Minneapolis, MN, USA) or monoclonal mouse anti-human HMGB1 IgG2b (R&D Systems) and biotinylated anti-goat IgG or anti-mouse IgG secondary antibodies (Vector Laboratories, Burlingame, CA, USA). Immunoreactivity was amplified using biotin-avidin peroxidase conjugates (Vectastain ABC kit, Vector Laboratories). 3,3′-diaminobenzidine was used as chromogen (DAB Peroxidase substrate kit, Vector Laboratories). Counterstaining was performed using Mayer’s hematoxylin. Slides were dehydrated with ethanol: 1×95% for 1 min, 1×100% for 6 min and cleared in n-Butanol before mounting (Pertex Mounting Media, Leica Microsystems, Germany).

Immunohistochemistry for representative markers of hematopoietic precursor cells CD34, the intermediate filament vimentin and smooth muscle α-actin (α-SMA) were performed on adjacent sections of PEA specimens using the automated Ventana Benchmark platform (Ventana Medical Systems, Tucson, AZ, USA) according to routine protocols of the clinical institute of pathology. Sections were stained with monoclonal mouse anti-human α-SMA (Clone1A4; Dako, Denmark, Europe), monoclonal mouse anti-human CD34 IgG_1_ (Clone QBEnd/10; Novocastra, Leica Biosystems Newcastle, UK, Europe) and monoclonal rabbit anti-human vimentin IgG (clone SP20; Thermo Fisher Scientific, Fremont, USA). Heat pre-treatment was conducted in Ultra cell conditioner number 1 buffer (Ultra CC1; pH 6). Color was developed with Ultraview Universal Detection DAB-kit (Ventana Medical Systems). Immunohistochemical staining for RAGE and HMGB1 was reproduced with the described automated system.

Omission of primary antibody served as negative control. Hematoxylin and eosin (H&E) staining was performed according to routine protocols. Analysis and image documentation was done with Axio Imager 2 microscope and AxioVision software (Carl Zeiss International, Germany).

### Grading of pulmonary vascular lesions

Specimens of explanted lungs from patients undergoing lung transplantation for iPAH were harvested at the time of transplantation. Sections were stained for hematoxylin and eosin (H&E) and Elastica van Gieson (EvG) to visualize elastin and analyzed according to a modified Heath Edwards classification system changes in small pulmonary arteries. Grade 1 is characterized by extension of muscle cells into distal arterioles and thickening of the media of muscular arteries. Grade 2 is defined as hypertrophy of the media with intimal proliferation in small muscular arteries. Grade 3 shows progressive fibrous vascular occlusion and concentric intimal fibrosis. Grade 4 is characterized by progressive arterial dilatation with plexiform lesions, grade 5 by chronic dilatation with fibrosis of intima and media, prominent plexiform and angiomatoid lesions and pulmonary hemosiderosis.

### Evaluation of immunoreactivity

Analysis of immunoreactivity was performed by two observers blinded to the type of antibodies used for staining. Two to four slides per patient were assessed. We assigned a score from 0 to 3 to assess staining intensity for RAGE or HMGB1 cytoplasmic or nuclear expression in PEA specimens, PA and small PA in lungs of patients with pneumothorax, iPAH and COPD (0, no staining; 1, weak; 2, moderate; 3, strong).

### Detection of serum proteins

To test the hypothesis that RAGE and HMGB1 are involved systemically in patients with pulmonary hypertension, we employed enzyme-linked immunosorbent assays (ELISA) for the detection of sRAGE, esRAGE, S100A9 and HMGB1 in serum of patients with CTEPH, iPAH, AVS and healthy volunteers. All ELISA tests were performed according to the manufacturers’ instructions: sRAGE (RAGE Duoset Elisa, RnD Systems, Minneapolis, MN, USA), esRAGE (B-Bridge International Inc., CA, USA), S100A9 (Abnova, Taipei City, Taiwan) and HMGB1 (IBL International GmbH, Hamburg, Germany). Researchers performing the assays and data analyses were blinded to the groups associated with each sample.

### Statistical methods

We performed an observational study with longitudinal (cohort study: measurements before and after PEA, LuTX and AVR) and cross-sectional design (e.g. serum sRAGE concentration in CTEPH/iPAH compared to controls). Statistical analysis of data was performed using SPSS software (version 20; IBM SPSS Inc., IL, USA). Data were reported as mean (median) ± standard deviation (and standard error mean) in tables and as mean ± standard error mean in the abstract and results section. The concentrations of proteins in serum of patients with CTEPH, iPAH and AVS were compared to those of healthy volunteers using independent Student’s t test or One-way ANOVA for normal (Gaussian) distributions. Kruskal-Wallis rank test or Mann-Whitney U test was used to evaluate non-normal distributions. Post hoc comparisons were computed with the Tukey correction. The paired t-test was applied to before and after measurements made on the same group of subjects, such as sRAGE serum concentrations before and after PEA. Pearson’s χ2 test for independence was used for analysis of categorical data, such as sex differences. Spearman’s rank correlation test was used to assess possible correlations of sRAGE and mean pulmonary artery pressure. The level of statistical significance was set at <0.05 (two-tailed p-values).

## Results

### Expression of RAGE and HMGB1 in endarterectomized tissue from CTEPH patients, regular PA morphology and diseased small PAs in patients with iPAH

#### Expression of RAGE and HMGB1 in endarterectomized tissues of patients with CTEPH

**Figure 1:**
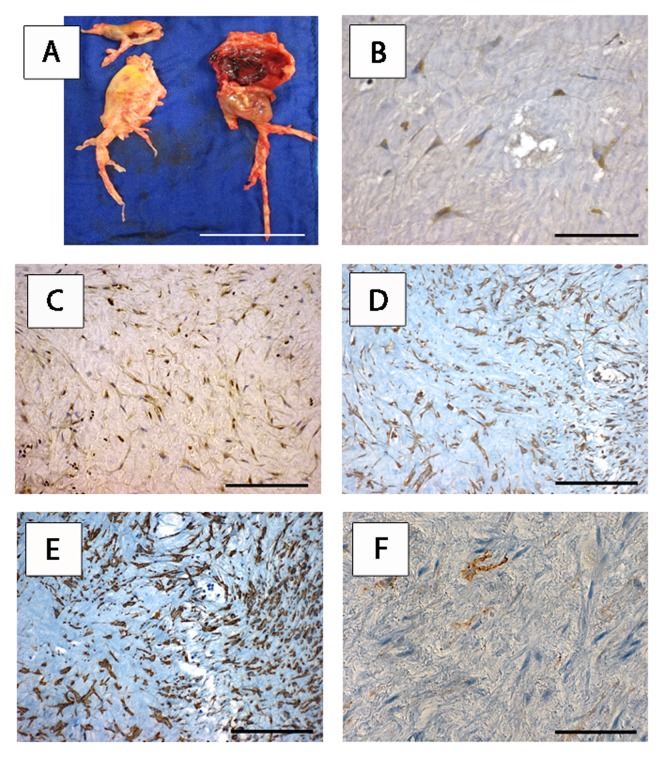
RAGE and HMGB1 are expressed in myofibroblasts of endarterectomised chronic thromboembolic tissues of CTEPH patients. One representative patient is shown (12 out of 15 patients (80%) displayed analogous staining patterns. Photograph showing the macroscopic aspect of a representative PEA specimen (A). Scale bar: 6 cm. Immunohistochemical expression of RAGE (B, scale bar: 20 µm), HMGB1 (C), vimentin (D), alpha-smooth muscle actin (E) and CD34 (F) on adjacent tissue sections of the PEA specimen shown in (A). Scale bar: 40 µm. *RAGE* receptor for advanced glycation endproducts, *HMGB1* high mobility group box 1, *CTEPH chronic thromboembolic pulmonary hypertension, PEA* pulmonary endarterectomy.

Diseased central, lobar and segmental PAs in CTEPH patients undergoing PEA showed thromboembolic material incorporated into the remodeled vessel wall in the form of intimal thickening and formation of neointima. The macroscopic aspect of a representative PEA specimen is shown (Fig. 1A). Hematoxylin and eosin stained sections revealed the prototypic morphology of fibroblasts/fibrocytes forming a honeycomb-like network. To test the hypothesis that RAGE and HMGB1 are involved in CTEPH, we employed immunohistochemical analysis for the detection of RAGE and HMGB1 in PEA specimens (fig. 1B+C). We found cytoplasmic staining for RAGE and cytoplasmic and nuclear staining for HMGB1 in the majority of patients examined (12 out of 15 patients, 80.0%). The specimens of the same 12 patients stained for RAGE and HMGB1. Nuclear staining for RAGE was not detected. In positive specimens 70.9±4.2% of cells showed RAGE and 72.8±4.6% showed HMGB1 expression.

#### Identification of RAGE and HMGB1 expressing cells as myofibroblasts

We performed analysis on adjacent sections to further characterize RAGE^+^ and HMGB1^+^ cells in endarterectomized tissues. We found that RAGE^+^ and HMGB1^+^ cells were also expressing the intermediate filament vimentin and α-SMA. CD34, a representative marker of hematopoietic precursor cells, did not correlate with the expression pattern seen for RAGE^+^ HMGB1^+^ vimentin^+^ α-SMA^+^ cells. The expression of CD34 expressing cells can rather be described as sporadic nests in 30.0% of tissues, a homogeneous distribution throughout any of the specimens was not found (fig. 1).

#### RAGE and HMGB1 expression in neointima and small vessel-like structures recanalizing distal “myofibrotic” clots

**Figure 2:**
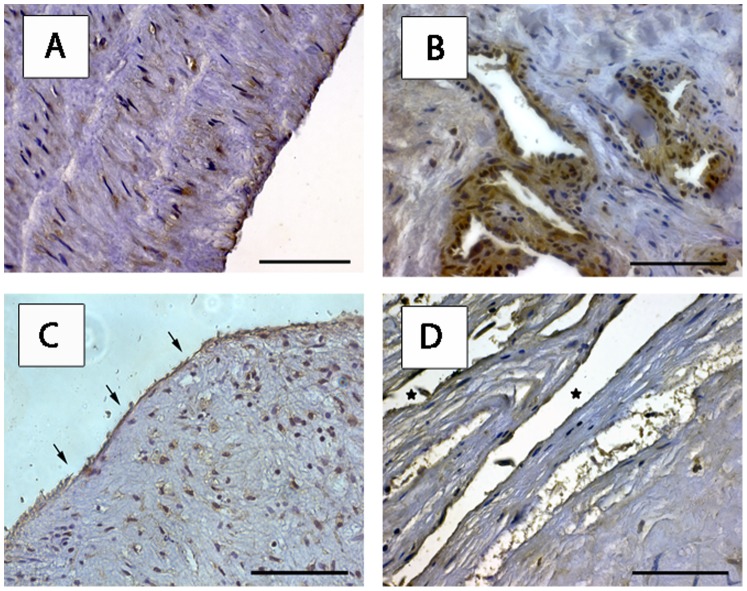
RAGE and HMGB1 expression on endothelial cells of regular PA and endarterectomised tissues. Representative examples of 12 examined patients are shown. Immunohistochemical analysis of regular main pulmonary artery with RAGE expression on endothelium and smooth muscle cells is shown (A). Scale bar: 40 µm. RAGE expressing endothelial cells in vessel-like structures recanalizing the matrix of distal PEA material (B). Endothelial cells expressing RAGE in proximal PEA tissue (arrows point at neointima, C) and HMGB1 (* in recanalizing vessel-like structures, D) in distal PEA specimen are displayed. Scale bar: 20 µm. *RAGE* receptor for advanced glycation endproducts, *HMGB1* high mobility group box 1, *PA* pulmonary artery, *PEA* pulmonary endarterectomy.

RAGE cytoplasmic staining was detected in endothelium of the intimal vessel wall (100% of endothelial cells) and smooth muscle cells of the media (64.8%) of regular main PAs. Vessel-like structures in distal areas of endarterectomized tissues showed RAGE and HMGB1 expression. Neointima (cell layer outlining the luminal surface) covering the organized thromboembolic material of diseased PAs displayed RAGE and HMGB1 expression (fig. 2). These staining patterns were analogous in all 12 patients examined.

#### Differentiated expression of RAGE in small PAs (<500 µm in diameter)

**Figure 3:**
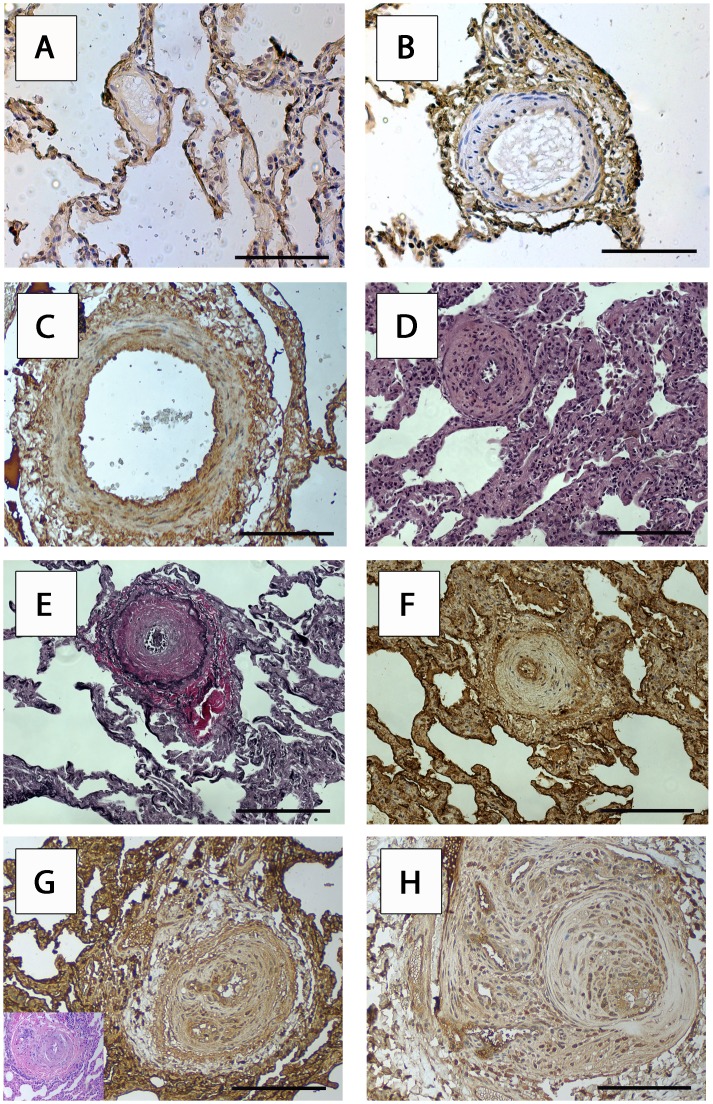
RAGE expression in pulmonary vascular changes of patients with iPAH. Representative examples of immunohistochemical analyses of pathognomonic lesions in lung of patients with iPAH according to the modified Heath Edwards classification in PA vessels smaller than 500 µm in diameter are shown (3 patients per every Heath Edwards group were analysed). RAGE expression in (A) a morphologically regular small PA - stage 0 and (B) a stage 1 histological change in lung of a patient operated for pneumothorax (COPD 0, centriacinar emphysematous changes). RAGE expression in characteristic stage 2 changes in a lung of a patient with iPAH (C). Scale bar in A, B and C: 80 µm. Adjacent sections of H&E (D) and EvG (E) and RAGE staining (F) for stage 3 changes in iPAH. Scale bar in D, E and F: 40 µm. Stage 4, angiomatoid (insert with adjacent H&E section, G), and stage 5, plexiform (H) PA vessel changes are shown. Scale bar in G and H: 80 µm. *iPAH* idiopathic pulmonary arterial hypertension, *PA* pulmonary artery, *RAGE* receptor for advanced glycation endproducts, *COPD* chronic obstructive pulmonary disease, *H&E* hematoxylin and eosin staining, *EvG* Elastica van Gieson staining.

In order to quantify the expression of RAGE in PA changes prototypical for PH we employed immunohistochemical analysis for the detection of RAGE in lung tissue specimens of patients with iPAH, COPD and pneumothorax (fig. 3). Prototypical Heath Edwards lesions were identified by H&E and EvG staining. We investigated lesions from three patients for every Heath Edwards group. Cytoplasmic RAGE expression was found in endothelium of Heath Edwards stages 0–5. The staining intensity in endothelial cells was as follows: stages 0–1: weak, stages 2–5: moderate. In smooth muscle cells of small muscular PAs RAGE was detectable with the following staining intensities: stages 0–1 (absent to weak), stages 2–5 (weak to moderate). Nuclear staining for RAGE was not detected. The described staining patterns and intensities were uniform throughout all patients examined.

### Systemic measurements: Concentration of sRAGE, esRAGE and RAGE ligands: HMGB1 and S100A9 in serum of patients with PH and AVS

#### Increased levels of sRAGE, esRAGE and HMGB1 in serum of patients with CTEPH

**Figure 4:**
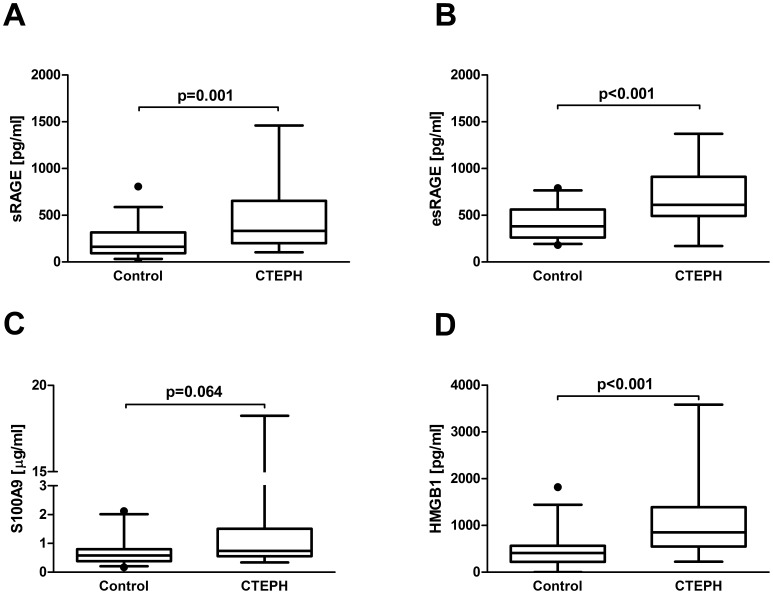
Serum concentrations of RAGE axis molecules in patients with CTEPH. Box plot analysis of serum concentrations of sRAGE (A), esRAGE (B), S100A9 (C) and HMGB1 (D) in patients with CTEPH (n = 26) and controls (n = 33). Independent Student’s t-test was used to compare groups. *RAGE* receptor for advanced glycation endproducts, *sRAGE* soluble RAGE, *esRAGE* endogenous secretory RAGE, *S100A9* member of S100 family of Ca+ binding proteins, *HMGB1* high mobility group box1, *CTEPH* chronic thromboembolic pulmonary hypertension.

Basic demographic and hemodynamic data of patients with CTEPH and volunteers are detailed in table 1. There was no statistically significant difference in age (p = 0.828) and sex (p = 0.985) between patients with CTEPH (n = 26) and controls (n = 33). We found significantly elevated serum concentrations of sRAGE in patients with CTEPH compared to controls (sRAGE [pg/ml] 467.2±72.6 vs.198.6±28.3; p = 0.001). Similarly, serum concentrations of the splice variant esRAGE were significantly higher than those of controls (esRAGE [pg/ml] 703.7±63.1 vs. 414.5±31.8; p<0.001). The RAGE ligand S100A9 was not significantly different (S100A9 [µg/ml] 2.1±0.8 vs. 0.7±0.09; p = 0.064) whereas HMGB1 was significantly elevated in serum of patients with CTEPH (HMGB1 [pg/ml] 1141.1±173.1 vs. 464.3±66.6; p = 0.001; fig. 4, table 1). There was no significant correlation of serum sRAGE concentrations with mean pulmonary artery pressure in patients with CTEPH (correlation coefficient 0.116, p = 0.646).

#### Higher concentrations of sRAGE and esRAGE, but not HMGB1 and S100A9 in patients with iPAH

**Figure 5:**
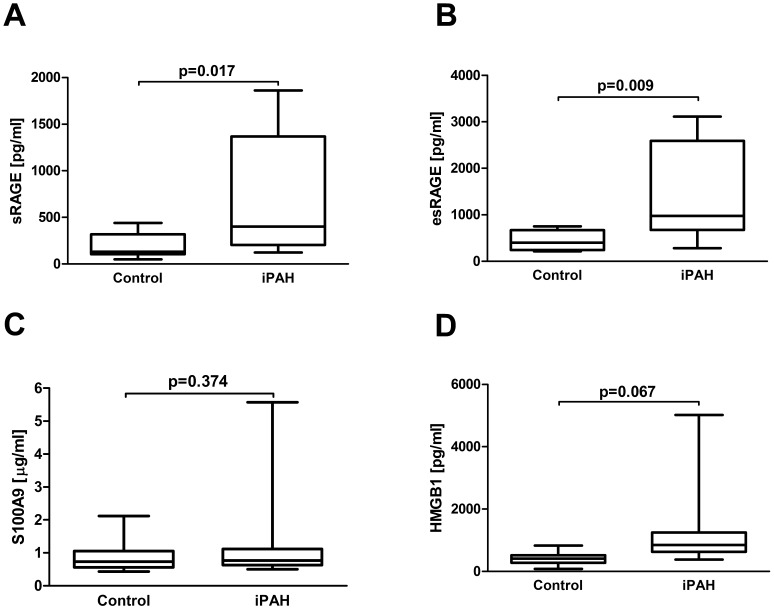
Serum concentrations of RAGE axis molecules in patients with iPAH. Box plot analysis of serum concentrations of sRAGE (A), esRAGE (B), S100A9 (C) and HMGB1 (D) in patients with iPAH (n = 8) and controls (n = 11). Independent Student’s t-test was used to compare groups. *RAGE* receptor for advanced glycation endproducts, *sRAGE* soluble RAGE, *esRAGE* endogenous secretory RAGE, *S100A9* member of S100 family of Ca+ binding proteins, *HMGB1* high mobility group box1, *iPAH* idiopathic pulmonary arterial hypertension.

Basic demographic and hemodynamic data of patients with iPAH and volunteers are detailed in table 2. There was no significant difference in age (p = 0.963) and sex (p = 0.381) between patients with iPAH and controls. We found significantly elevated concentrations of sRAGE and esRAGE in patients with iPAH (sRAGE [pg/ml]: 743.7±254.3 vs. 195.5±39.5; p = 0.017; esRAGE [pg/ml] 1391.1±379.4 vs. 423.2±59.4; p = 0.009). Conversely, the measured RAGE ligands: serum S100A9 and HMGB1 in patients with iPAH did not differ from controls (S100A9 [µg/ml] 1.4±0.6 vs. 0.9±0.1, p = 0.374; and HMGB1 [pg/ml] 1419.4±610.1 vs. 415.1±65.5, p = 0.067; fig. 5, table 2). There was no significant correlation of serum sRAGE concentrations with mean pulmonary artery pressure in patients with iPAH (correlation coefficient −0.144, p = 0.734).

#### Serum concentrations of sRAGE were significantly higher in iPAH and CTEPH (iPAH>CTEPH) in relation to reduced concentrations in AVS patients

We wanted to test the hypothesis that sRAGE serum concentrations are only elevated in diseases of the pulmonary circulation leading to pressure overload of the right ventricle compared to disease leading to pressure overload of the left ventricle, such as AVS. Basic demographic and hemodynamic data of patients with CTEPH undergoing PEA, iPAH undergoing lung transplantation, AVS undergoing AVR and volunteers are detailed in table 3.

**Figure 6:**
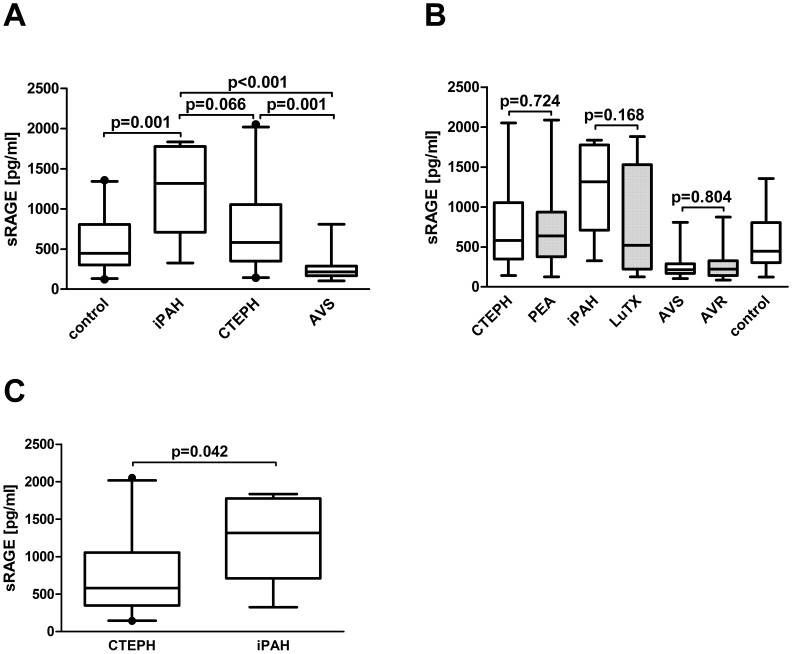
Serum concentrations of sRAGE in patients before and after surgery for CTEPH, iPAH and AVS. Box plot analysis of serum concentrations of sRAGE in patients with CTEPH (n = 20), iPAH (n = 7), AVS (n = 15) and controls (n = 28, A). Box plot analysis of sRAGE in serum of patients with CTEPH before and after PEA, in patients with iPAH before and after double lung transplantation and in patients with AVS before and after AVR (B). Box plot analysis of serum concentrations of sRAGE in patients with CTEPH and iPAH (C). One-way ANOVA was used to compare groups. Post hoc comparisons were computed with the Tukey correction. *RAGE* receptor for advanced glycation endproducts, *sRAGE* soluble RAGE, *CTEPH* chronic thromboembolic pulmonary hypertension, *PEA* pulmonary endarterectomy, *iPAH* idiopathic pulmonary arterial hypertension, *AVS* aortic valve stenosis, *AVR* aortic valve replacement.

*ANOVA* analysis of sRAGE concentrations from patients with CTEPH, iPAH, AVS and healthy volunteers revealed significant differences (p<0.001). *Post-hoc* comparisons showed significantly higher serum concentrations of sRAGE in patients with iPAH (p<0.001) and CTEPH (p = 0.001) compared to AVS (fig. 6). Further *post-hoc* comparisons revealed no difference in serum sRAGE concentration between patients with CTEPH and iPAH (p = 0.066). Separate analysis of patients with CTEPH and iPAH showed significantly higher serum sRAGE concentrations in patients with iPAH compared to CTEPH (independent samples t-test: p = 0.042). Serum sRAGE concentrations were significantly reduced in patients with AVS compared to controls (p = 0.001).

#### Influence of surgical therapy on serum sRAGE concentrations

There were no significant differences in sRAGE serum concentrations before and after surgical therapy for CTEPH, iPAH or AVS. Basic demographic and hemodynamic data are detailed in table 3. The results were as follows: CTEPH patients before and after PEA (sRAGE [pg/ml] 743.29±26.98 vs. 688.70±88.68, p = 0.724), iPAH patients before and after lung transplantation (sRAGE [pg/ml] 1216.0±213.5 vs. 772.8±262.6, p = 0.168) and patients with AVS before and after AVR (sRAGE [pg/ml] 260.2±44.2 vs. 274.4±51.4, p = 0.804; table 3, fig. 6).

For patients undergoing PEA, early (10 days after PEA) and late (one year after PEA) postoperative serum samples were available: There was no difference in sRAGE concentrations when measurements were stratified between early and late: before and early after PEA (n = 12): (sRAGE [pg/ml]: 692.8±168.3 vs. 534.1±59.8, p = 0.451; before and one year after PEA (n = 9): 804.9±214.7 vs. 877.6±167.1, p = 0.767; in comparison to all patients (n = 21) together: as above: p = 0.724.

## Discussion

The results of this study describe for the first time the expression of RAGE and HMGB1 in myofibroblasts of patients with CTEPH. Prior studies have shown the majority of cells in endarterectomised tissues from patients with CTEPH to be myofibroblasts [46], [47]. The presence of multipotent mesenchymal progenitor cells (capable of adipogenic and osteogenic differentiation) was described [46]. Further, endothelial progenitor cells (CD34^+^CD133^+^Flk-1^+^) were identified in neointima of proximal thromboembolic material as well as distal regions (downstream of the thromboembolic material) [47]. Myofibroblast-like cells were described as hyperproliferative, anchorage-independent, invasive and serum-independent [48]. These myofibroblast-like cells were later termed sarcoma-like cells as the injection into the tail veins of C.B-17/lcr-scid/scidJcl mice led to the development of tumors growing along the intimal surface of the pulmonary vessels (a mouse model for pulmonary artery intimal sarcoma) [49]. A possible functional role of RAGE and HMGB1 in myofibroblasts of remodeled PA vessels in patients with CTEPH may be inferred from recent studies describing a role for RAGE in iPAH. In PASMCs of patients with PAH, RAGE was 6-fold upregulated, induced STAT3 activation and decreased the expression of BMPR2 and PPARγ. The described cell phenotype could be induced by RAGE agonist 100A4 in control PASMCs and reversed by RAGE blockade with RAGE small interfering RNA (siRNA) in both cell types. RAGE blockade reduced PA pressures and right ventricular remodeling associated with improved lung perfusion and vascular remodeling in *in vivo* animal models of monocrotaline- and Sugen-induced PAH. Immunofluorescence staining revealed a correlation of RAGE protein expression with disease severity in patients with PAH [42]. Disease severity was classified as mild, moderate and severe. As increased sRAGE concentrations in our study did not correlate with PAP*mean*, we chose to investigate RAGE expression in prototypical vessel changes according to the modified Heath and Edwards classification that was routinely applied during the diagnostic workup of lungs with iPAH at our institution. We found a greater staining intensity in endothelial cells as well as smooth muscle cells of higher Heath Edwards grades.

Recently, a role of the damage-associated molecular pattern molecule (DAMP) HMGB1 was shown to contribute to PH via a TLR4-dependent mechanism in a mouse model of CH-induced PH. In patients with iPAH extra-nuclear HMGB1 in pulmonary vascular lesions was identified. Increased concentrations of serum HMGB1 correlated with PAP*mean*. In C57BL6/J mice exposed to CH-induced PH a statistically not significant nearly two-fold increase in RAGE mRNA was observed. Also of interest are the observations in RAGE knockout (RAGE^−/−^) compared to wild-type mice exposed to CH: there was the same increase in right ventricular (RV) systolic pressure, but decreased RV hypertrophy in RAGE^−/−^ mice. In the same model RAGE^−/−^ mice neither showed significantly different vascular changes nor did the levels of mouse endothelin −1 (ET-1) or mouse soluble intracellular adhesion molecule 1 (sICAM-1) differ [43]. Our data on human patients support a role of the RAGE axis, namely RAGE and HMGB1, in iPAH. The lack of significant differences in pulmonary vascular changes and circulating cytokines in RAGE^−/−^ mice is puzzling and does not parallel our data – stronger expression of RAGE in higher Heath Edwards stages - or the above described data by Meloche et al - 6-fold upregulation of RAGE in PASMCs of patients with PAH [42]. With the current limited evidence we can only attribute the differences between mice and men to intrinsic mechanisms of the mouse model of CH-induced PH.

The migration of human pulmonary artery endothelial cells (huPAEC) in vitro could be inhibited by HMGB1 via TLR4 and IRF3-dependent mechanisms [50]. If this HMGB1 effect can also be reversed by blockade of RAGE still has to be tested. In our study we demonstrated RAGE expression in endothelial cells of large and small (<500 µm) regular PAs, neointima of proximal remodeled PAs and recanalizing vessel-like structures of distal endarterectomised tissue of patients with CTEPH, as well as prototypical Heath Edwards lesions in patients with PH. Endothelial RAGE was present in health and disease. The behavior of endothelial cells from diseased and healthy tissues could reveal further information.

In patients with chronic heart failure and impaired left ventricular function activation of the immune system as measured by increased levels of proinflammatory cytokines is associated with poor prognosis [51], [52]. No differences in serum concentrations of the measured cytokines: TNF-alpha, its soluble receptors 1 and 2 (sTNFR1 and 2), IL-10, high sensitivity C-reactive protein (hsCRP) and N-terminal-pro-B-type natriuretic peptide (NT-proBNP was measured in plasma) were found when right ventricular dysfunction due to CTEPH and left ventricular dysfunction due to chronic heart failure were compared [51]. In order to help untangle the possible sources by which sRAGE serum concentrations increased in patients with CTEPH and iPAH we compared serum measurements in patients with CTEPH before and after PEA and in patients with iPAH before and after lung transplantation to those of patients with AVS before and after AVR. In this experiment right ventricular remodeling with pressure overload resulting from pulmonary vascular disease (CTEPH and iPAH) is compared to left ventricular remodeling as a consequence of the pressure-overloaded left ventricle observed in patients with aortic stenosis. The results of our experiment point to a pulmonary source of sRAGE as there was no elevation in serum of patients with AVS. A normalization of sRAGE in serum after PEA or lung transplantation can probably not be expected regarding the neurohumoral and immunological disturbances occurring in these patients [10]. Possible pitfalls of our model are two emerging conceptual differences between right and left ventricular adaptation and remodeling: (1) right ventricular enlargement occurs earlier in the course of PAH when compared to pressure-overloaded left ventricles, probably because of the smaller thickness of the right ventricle that will experience greater wall stress for comparable increases in pressure. And second, there is much less myocardial fibrosis in patients with RV pressure overload compared to patients with AVS which explains the high rate of recovery of right ventricular function after lung transplantation, even when right ventricular ejection fraction was severely reduced at the time of transplantation [53], [54], [55]. Regarding these differences, we cannot exclude the possibility that sRAGE is derived (in part) from a myocardial source. Concerning the myocardium, the intra-coronary administration of sRAGE attenuated cardiac remodeling and fibrosis in minipigs with ischemia-reperfusion injury [56].

Our results on patients with AVS are in line with a previous study that showed that plasma sRAGE levels were significantly lower in patients with AVS than in controls and independently associated with the risk for AVS. In that study there was an inverse correlation with age, cholesterol levels and coronary calcification [57].

In lung transplant recipients elevated plasma sRAGE concentrations measured four hours after reperfusion of the lung allograft were associated with longer duration of mechanical ventilation and longer intensive care unit length of stay [58]. Increased plasma levels of sRAGE were associated with primary graft dysfunction at six and 24 hours after lung transplantation [59]. Elevated plasma sRAGE measured 24 hours postoperatively was associated with the development of bronchiolitis obliterans syndrome [60]. No data exist on RAGE and lung transplantation for iPAH. In our study there was a non-significant reduction in serum sRAGE concentrations in stable lung transplant recipients 3 weeks post transplantation.

Multivariate logistic regression analysis revealed plasma sRAGE concentrations immediately after cardiopulmonary bypass surgery to be an independent predictor for postoperative acute lung injury after cardiac surgery in children [61]. Similarly, S100A12 and sRAGE were associated with increased length of hospitalization after non-urgent coronary artery bypass grafting surgery [62]. The comparison of early (ten days) and late (one year) sRAGE measurement in our study did not show significant differences which infers that the high serum concentrations also after surgery may not only be influenced by the trauma of the surgical intervention alone but also by disease specific alterations in the RAGE axis that may not be influenced by current treatment modalities.

Current standard preoperative evaluation of PEA candidates is unreliable in predicting patients at risk for persistent pulmonary hypertension because of surgically inaccessible thromboembolic material or coexistent small vessel disease which are major reasons for poor outcome [63]. Attempts to identify high risk patients are currently investigated. In a recent study, the preoperative assessment of upstream resistance correlated with postoperative pulmonary resistance index and PAP*mean* [64]. In our study, preoperative sRAGE serum concentrations were significantly higher in patients with iPAH compared to CTEPH and did not correlate with the height of pulmonary artery pressures. This could have implications on the decision to perform pulmonary endarterectomy on patients with CTEPH. The question that has to be answered in future studies is: can high serum concentrations of sRAGE, such as measured in iPAH in this study, unmask distal disease that is not accessible to PEA and thus be of value in preoperative decision making regarding operability of CTEPH patients with high pulmonary vascular resistance (PVR>1200 dynes.cm/s^5^) [65]?

As sRAGE serum concentrations did not correlate with their corresponding pulmonary artery pressures in this study we can only hypothesize about an on/off-phenomenon of chronic inflammation in iPAH and CTEPH patients. The current information raises new questions. What pathophysiologic threshold has to be reached to turn on chronic inflammation through the RAGE axis? Is the RAGE axis involved in the primary events of remodeling of the thromboembolic material into the PA vessel wall or is it turned on at later stages of CTEPH and iPAH? Could RAGE blockade terminate chronic inflammation in these diseases and be of clinical value in patients as an adjunct to current therapies?

We are not suggesting that our absolute concentration values can be used to make any judgments about the diagnosis of, for example CTEPH. While comparative results (e.g. control vs. CTEPH) gained during one experiment could be repeated in separate ELISA experiments, the absolute values for the individual serum samples vary in our experienced hands with the recommended additional reagents from the manufacturer. So we never compare absolute values from samples measured with the RAGE Duoset from different experiments. The intraassay coefficient of variation was 2.3%. We run control serum samples on each ELISA. The RAGE Duoset has quite some interassay variability. The commercially available ELISA is sold for research use only and not for diagnostic purposes. We don’t see this interassay variability with the other ELISA assays used in this manuscript.

Soluble RAGE was measured in serum/plasma of other pulmonary diseases with different methods. In stable COPD patients plasma sRAGE was significantly lower compared to healthy control subjects: 400.2 pg/ml vs. 783.3 pg/ml, p<0.001; measured by ELISA, R&D systems, Minneapolis, MN, USA [66]. Another study used two different multiplex platforms (Luminex multi-analyte profiling at Rules Based Medicine, RBM, Austin, TX and Searchlight at Aushon Biosystems, Bellaria, MA) to find significant differences in serum sRAGE concentrations in non-smokers, smokers, COPD I/II and COPD III/IV: median sRAGE values [ng/ml] 4.2, 3.2, 2.7 and 2.2, p = 0.003 [67]. A study using Quanitkine human RAGE ELISA kit (R&D systems, Minneapolis, MN, USA) found significantly different lower sRAGE concentrations in patients with COPD compared to smoking and nonsmoking controls subjects: sRAGE values [pg/ml]: 1351.1 vs. 1736.6 and 1797.3, p<0.001 [68]. There are 2.8- to 10.5-fold differences between the controls or COPD patients when the three studies with different analytical methods and different population samples are compared. Regarding the different absolute concentrations reported for sRAGE as exemplified with three studies for COPD as another pulmonary disease, it is too vague for us to draw conclusions from the comparison of absolute sRAGE measurements between our and other studies.

In summary, we have shown the expression of RAGE and HMGB1 in myofibroblasts of endarterectomised tissues from patients with CTEPH and increased expression of RAGE in prototypical lesions in lung of patients with iPAH. Our immunohistochemical results were corroborated by alterations in the serum concentration of soluble RAGE variants and HMGB1. The results may have substantial implications for diagnosis and/or treatment of patients with pulmonary hypertension. PEA improves the local control of disease with the resultant decrease in pulmonary artery pressure but may not influence the systemic inflammatory mechanisms in CTEPH patients through the RAGE pathway. A more detailed understanding of the RAGE-HMGB1 axis and related molecules in diseases associated with pulmonary hypertension is needed and warrants future study.
